# Acute Myocardial Infarction Secondary to Triple Coronary Arteries Dissection in a Patient With Takayasu Vasculitis

**DOI:** 10.1016/j.jaccas.2023.101905

**Published:** 2023-06-12

**Authors:** Raad Tahtouh, Khaled Al Khodari, Jamila Al Ali Alhasan, Imad Awwad, Abdulrahman Arabi

**Affiliations:** a Internal Medicine Department, Hamad Medical Corporation, Doha, Qatar; b Cardiology Department, Hamad Medical Corporation, Doha, Qatar; c Internal Medicine Department, Hamad Medical Corporation, Doha, Qatar; d Radiology department, Hamad Medical Corporation, Doha, Qatar; e Cardiac Catheterization Laboratory, Cardiology Department, Hamad Medical Corporation, Doha, Qatar

**Keywords:** acute coronary syndrome, myocardial infarction, spontaneous coronary artery dissection, Takayasu arteritis

## Abstract

Synchronous spontaneous triple coronary artery dissection with Takayasu arteritis has not been previously reported in the literature. We describe the case of a young man with acute inferior wall myocardial infarction secondary to triple-vessel coronary artery dissection. Takayasu arteritis was diagnosed upon further investigation. (**Level of Difficulty: Intermediate.**)

## Central Illustration

**Figure:**
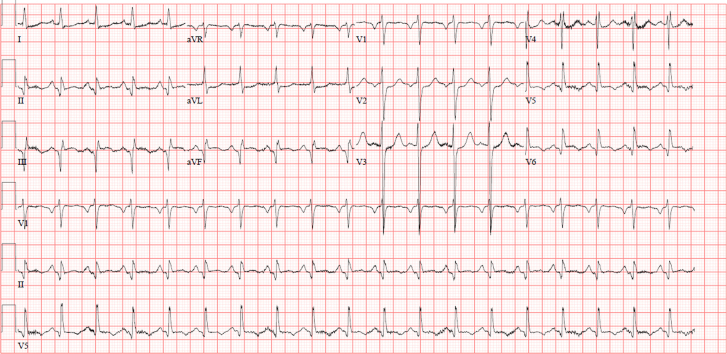

A 29-year-old man presented to the emergency department because of the sudden onset of progressive retrosternal chest pain for 2 hours. Initially, he was tachypneic and tachycardia, with a respiratory rate of approximately 35 breaths/min and a heart rate of approximately 120 beats/min. His blood pressure was 155/90 mm Hg, with no significant difference between his arms, Oxygen saturation was 92% on 2 L oxygen. The result of cardiovascular examination was unremarkable apart from fine bilateral basal crackles.

**Learning Objectives**

• To be able to make a differential diagnosis of coronary dissection causes in young patients.
• To understand the role of extracardiac causes in the development of coronary dissection.
• To be able to decide on acute dissection based on the expected underlying cause.

## Medical History

Our patient was previously healthy with no contributory medical history.

## Differential Diagnosis

Differential diagnoses included acute coronary syndrome (ACS), acute aortic dissection, and pulmonary embolism.

## Investigations

**Figure 1:**
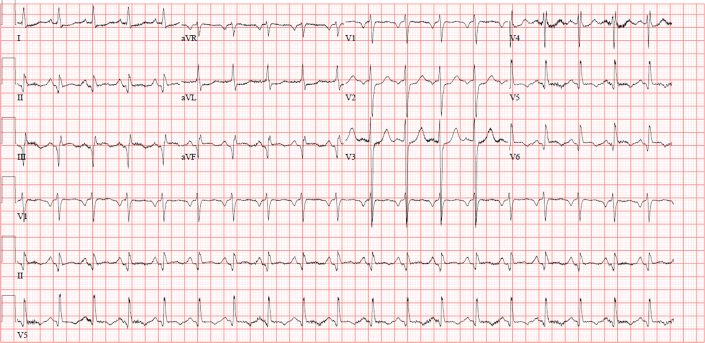
Electrocardiogram at Presentation Electrocardiogram showed sinus tachycardia with a heart rate of 120 beats/min, ST-segment elevation in leads II, III, and aVF along with reciprocal changes in leads I and aVL.

**Figure 2:**
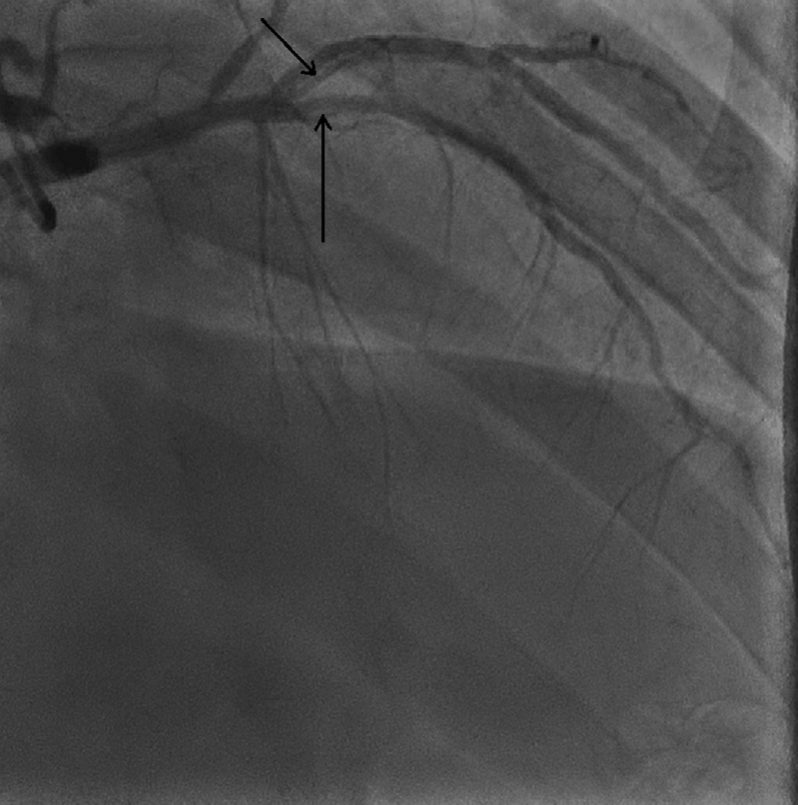
Coronary Angiogram Right anterior oblique cranial view of the left system revealed spontaneous coronary dissection in mid to distal left anterior descending coronary artery and diagonal 2 (D2).

**Figure 3:**
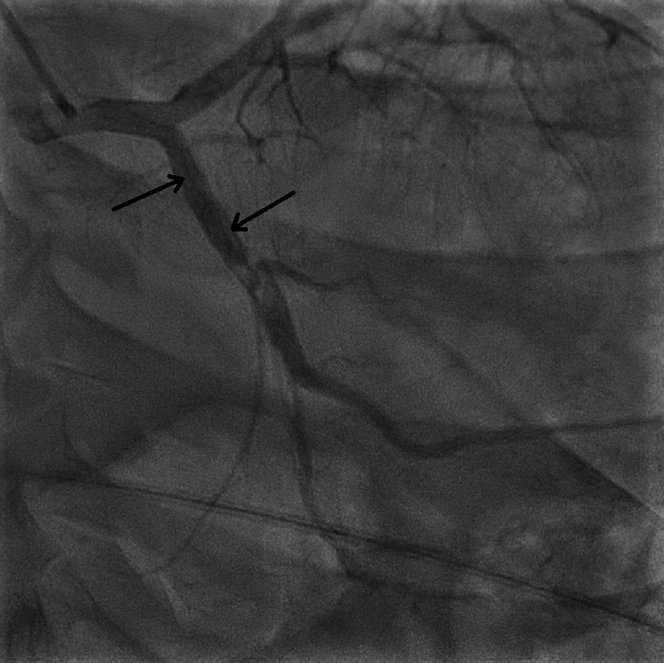
Coronary Angiogram Anteroposterior caudal view revealed a large filling defect from the proximal to distal left circumflex artery secondary to spontaneous artery dissection.

**Figure 4:**
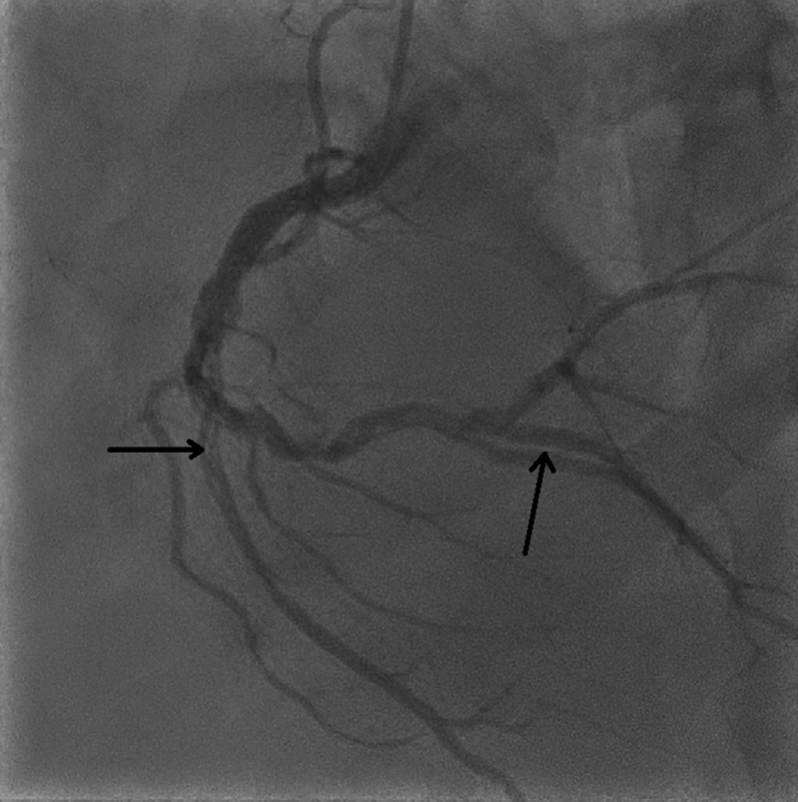
Coronary Angiogram Left anterior oblique cranial view of right coronary artery revealed longitudinal filling defects in mid to distal involving acute marginal branch secondary to spontaneous coronary artery.

An electrocardiogram (ECG) revealed ST-segment elevation (STEMI) with Q-wave in leads II, III, and aVF along with reciprocal ST-segment depression in leads I and aVL (Figure 1). A chest x-ray showed mild cardiomegaly with mild congestion. Blood test results showed leukocytosis (white blood count of 18,000/μL and a normal hemoglobin of 14.4 g/dL. In addition, he had an elevated troponin-t of 994 ng/L (normal ≤15 ng/L). which increased later to a maximum value of 4,842 ng/L; a high pro-BNP 1,990 (normal <300 pg/ml), and creatinine of 131/μmol. His electrolytes were within normal range, with mildly elevated alanine transaminase of 68 μ/L and aspartate aminotransferase of 114 μ/L. His lipid profile showed mildly elevated cholesterol of 5.8 mmol/L and low-density lipoprotein of 4.3 mmol/L, with triglycerides of 1.7 mmol/L. A coronary angiogram revealed spontaneous coronary artery dissection (SCAD) involving all coronary arteries, demonstrating longitudinal filling defects proximal to the distal left anterior descending artery (Figure 2, Video 1), the left circumflex artery (Figure 3), and the mid to distal right coronary artery (Figure 4, Video 2).

## Management

Because of the tachycardia without hypoperfusion, the impression was late presentation of inferior STEMI with Killip II classification. The patient was loaded with 300 mg of aspirin and 600 mg of clopidogrel, then moved to the catheterization laboratory for coronary angiography. A coronary angiogram revealed SCAD involving all coronary arteries.

Because the patient’s hemodynamic values were normal, the plan was to continue medical therapy. Subsequently, he was admitted to the cardiac intensive care unit for close monitoring. Echocardiography showed a markedly dilated left ventricle (LV) with severely reduced ejection fraction (22%) and no dissection in the ascending aorta; however, a large LV thrombus was detected in the apex. Transesophageal echocardiography confirmed the previous findings.

A repeated angiogram was considered in case of worsening ischemic symptoms. He was administered aspirin, clopidogrel, and intravenous heparin infusion for the LV thrombus as bridging therapy for warfarin to target a therapeutic INR.

There was no significant change in his creatinine level during his hospital stay; therefore, he was administered sacubitril with valsartan 24 mg/26 mg (Entresto) and dapagliflozin 10 mg sodium-glucose cotransporter-2 inhibitors (SGLT2).

The results of further investigations, including workups for autoimmune and connective tissue diseases, were unremarkable. He was discharged to use triple therapy for 1 month, then warfarin and clopidogrel for 1 year followed by warfarin alone. After 2 months, he presented with pain in the tips of all left-hand fingers that had persisted for 4 days. He stated that he had not been compliant with home medications. The INR was subtherapeutic, so an intravenous heparin infusion was started. The result of Doppler ultrasonography of the left upper limb was unremarkable.

**Figure 5:**
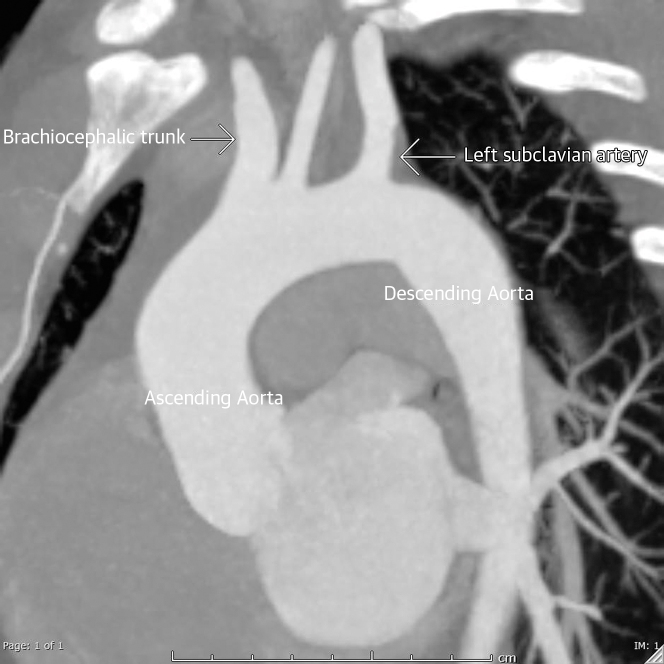
Computed Tomography Aortic Angiogram Findings were consistent with large vessel vasculitis, mainly Takayasu arteritis. Oblique sagittal maximum intensity projection image of the aortic arch showed focal eccentric mural thickening of the proximal left subclavian artery causing mild stenosis.

**Figure 6:**
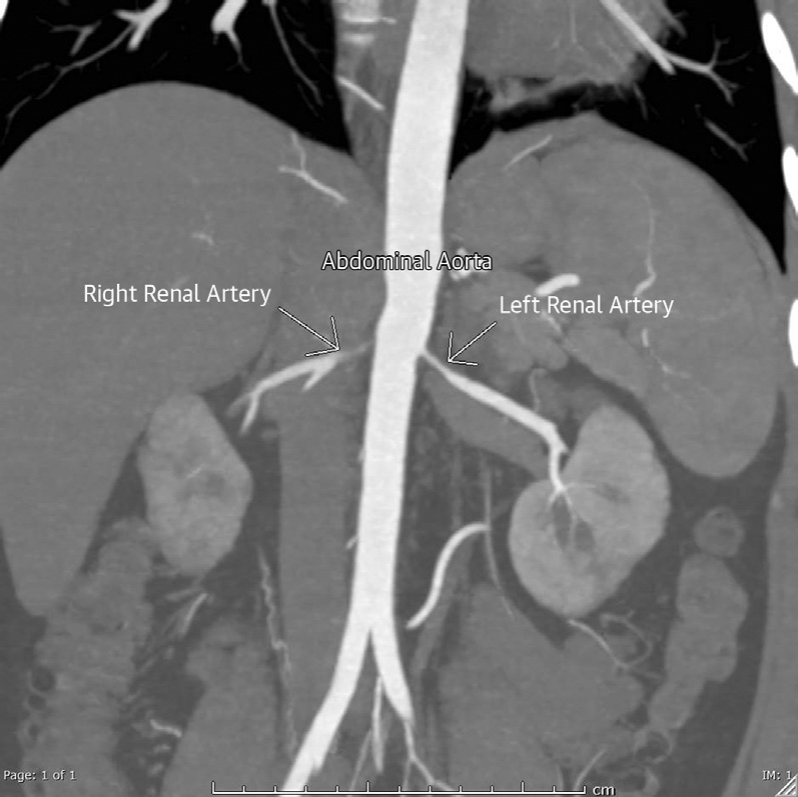
Coronal Maximum Intensity Projection Image Image of the abdominal aorta showed circumferential mural thickening of the proximal right renal artery and lower moiety left renal artery causing severe stenosis.

**Figure 7:**
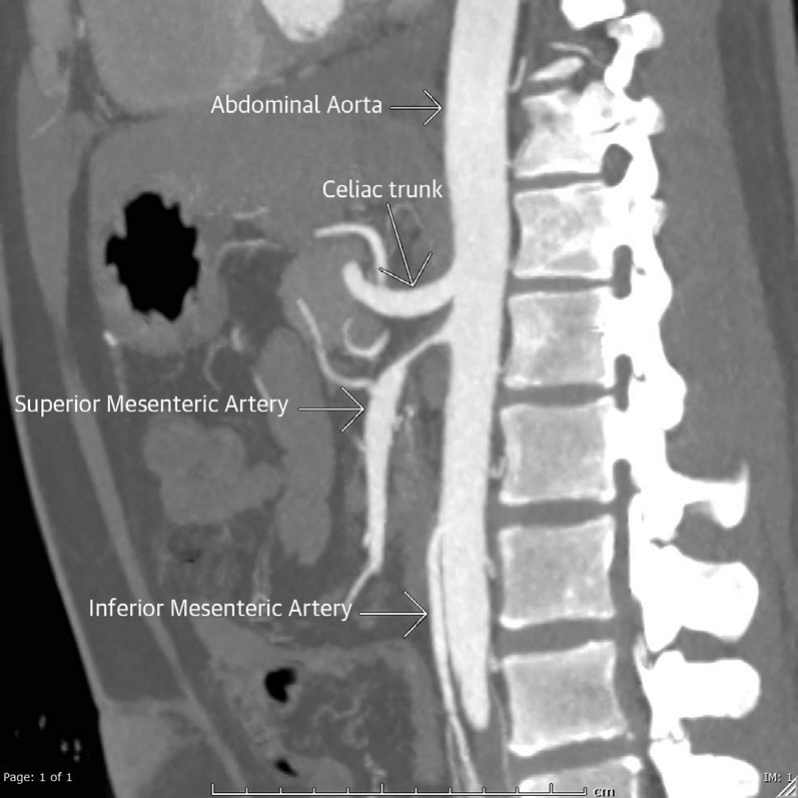
Sagittal Maximum Intensity Projection Image Image of the abdominal aorta circumferential mural thickening of the proximal superior mesenteric artery causing severe stenosis.

Persistent LV thrombus was found on repeated echocardiography. A CT angiogram was performed to examine the aortic arch and left subclavian and revealed concentric wall thickening with mild stenosis of the proximal left subclavian artery (Figure 5). There was a circumferential mural thickening of the proximal right renal artery and lower moiety left renal artery, causing severe stenosis (Figure 6), and circumferential mural thickening of the proximal superior mesenteric artery causing severe stenosis (Figure 7). The impression was large vessel vasculitis. Takayasu arteritis (TA) was the final diagnosis. Testing for autoimmune antibodies, hepatitis serology, inflammatory markers, and HIV gave negative results. He was administered intravenous methylprednisone 250 mg daily for 3 consecutive days, then oral prednisone 1 mg/kg/day. He was discharged with 60 mg prednisone daily along with vitamin D/calcium supplements. Through follow-up visits, he was doing well and was asymptomatic.

## Discussion

SCAD is a tear in the endothelial layer that may lead to intramural hematoma and subsequent interruption of blood flow.^1^ SCAD represents 1% to 4 % of all ACS cases, predominantly in female individuals. It is the most common cause of MI during pregnancy and accounts for 35% of ACS cases in women <50 years old.^1^

SCAD has unique causes and presentations, given that most affected patients are healthy young women without atherosclerotic risk factors. Furthermore, SCAD has been reported in multiple systemic inflammatory diseases such as systemic lupus erythematosus, polyarthritis nodosa, sarcoidosis, and cryoglobulinemia in the setting of hepatitis C.^1^ Similarly, fibromuscular dysplasia has been the most reported disease in association with SCAD.^1^

Multivessel SCAD is unusual and occurs in 9% to 23% of cases.^1^ Moreover, SCAD is rarely associated with systemic autoimmune inflammatory disease: 1.8% to 9 % as reported in different studies.^1^

A recent recommendation based on a case-control study was to avoid routine investigation for underlying autoimmune diseases in the setting of SCAD alone because inflammation is not the main process. Screening would be suggested when there is clinical suspicion when a patient has symptoms or physical examination findings suggestive of rheumatologic disease.^1^^,^^2^ However, our patient presented with SCAD, then with painful finger discoloration, which provided the guidance to proceed with CT angiography in a search for aortic-subclavian artery pathologic changes explaining the co-occurrence in 1 disease.

TA is a rare large vessel vasculitis that affects the aorta and its main branches. It presents with rheumatic symptoms and diminished peripheral pulses. The exact pathologic features are still unknown, but it affects female individuals more than male individuals.^3^

According to the American College of Rheumatology criteria, a diagnosis of TA is made by the presence of at least 3 out of 8 findings:^4^ 1) onset of the disease before the age of 40 years; 2) claudication of the extremities; 3) limitation in the brachial pulse; 4) pressure difference between the 2 arms >10-mm Hg; 5) presence of a murmur in the aortic area or subclavian artery; and 6) abnormal image findings. Our patient met 3 of these criteria by having a pressure difference of >10 mm Hg between the arms, an age of <40 years, and imaging confirmation of the abnormal findings typical of the condition.

TA has been reported as a rare cause of SCAD in left main artery dissection^5^ and the common carotid artery.^6^ Left main dissection was treated surgically, whereas carotid dissection was treated medically. Interestingly, triple SCAD was reported in Churg-Strauss syndrome.^7^ However, no case demonstrates diffuse and extended SCAD in TA arteritis.

Our patient received a diagnosis of triple SCAD and then eventually received a diagnosis of TA. We do not know the exact cause because all our information is based on case reports. It could be only a coincidence without direct interaction. However, thinking about systemic vasculitis should be kept in mind by clinicians who are investigating SCAD in an unusual presentation.

The management of SCAD is still controversial, and there is no current agreement on the best treatment options. It depends on the proportion of stenosis, hemodynamics, number of affected arteries, and available technical equipment.^1^

Medical treatment is efficient in most stable cases because it allows the healing process over time. Medical therapy helped in ≤97% of cases when an angiogram was repeated within weeks to months.^1^ Primary percutaneous coronary intervention in STEMI-SCAD is superior to conservative management. These days, there are no comparison studies for treatment outcomes between strategies of revascularization in ACS due to SCAD.^1^

According to the American Heart Association, conservative therapy is enough with patients in clinically stable condition and no high-risk anatomy lesions. However, urgent intervention with percutaneous coronary intervention or coronary artery bypass grafting would be recommended with high-risk anatomy lesions and active ischemia leading to instability.^1^

## Follow-Up

Our patient was treated medically for both diseases. We started with pulse steroid and then shifted to methotrexate for TA. The patient did not describe recurrent episodes of chest pain at rest or during exercise in the follow-up period. Repeated echocardiography was performed 1 month after the last admission and showed no evidence of LV thrombus.

## Conclusions

This case illustrates the unique presentation of triple SCAD leading to an ischemic heart attack secondary to underlying Takayasu vasculitis. We emphasize the importance of investigating possible systematic vasculitis diseases, even in the asymptomatic patient, as a cause of multivessel coronary dissection. Further research is required to improve the understanding of the underlying pathophysiology of TA accompanied by coronary artery dissection, assess the long-term treatment, and provide adequate follow-up and prevention strategies.

## Funding Support and Author Disclosures

The authors have reported that they have no relationships relevant to the contents of this paper to disclose.

## Appendix

Video 1 LAD Dissection Right anterior oblique (RAO) cranial view of the left system revealed spontaneous coronary dissection in mid to distal LAD and diagonal 2 (D2)

Video 2 RCA Dissection Left anterior oblique (LAO) cranial view of RCA revealed longitudinal filling defects in mid to distal involving acute marginal branch secondary to SCA.
